# Peri-Operative Anaphylaxis—An Investigational Challenge

**DOI:** 10.3389/fimmu.2019.01117

**Published:** 2019-05-29

**Authors:** Siraj A. Misbah, Mamidipudi Thirumala Krishna

**Affiliations:** ^1^Department of Clinical Immunology, Oxford University Foundation Hospitals NHS Trust, Oxford, United Kingdom; ^2^Department of Allergy and Immunology, University Hospitals Birmingham NHS Foundation Trust, Birmingham, United Kingdom; ^3^Institute of Immunology and Immunotherapy, University of Birmingham, Birmingham, United Kingdom

**Keywords:** peri-operative anaphylaxis, tryptase, skin tests, neuromuscular blockers, antibiotics, mast cell disorders, outcome of repeat anesthesia

## Abstract

Patients with suspected peri-operative anaphylaxis (POP) require thorough investigation to identify underlying trigger(s) and enable safe anesthesia for subsequent surgery. The changing epidemiology of POP has been striking. Previous estimates of the incidence of POP have ranged between 1:6,000 and1:20,000 anesthetics, but more recent data from France and the United Kingdom suggest an estimated incidence of 1:10,000. Other important changes include a change in the hierarchy of well-recognized triggers, with antibiotics (beta-lactams) supplanting neuromuscular blockers (NMB) as the leading cause of POP. The emergence of chlorhexidine, patent blue dye, and teicoplanin as important triggers have also been noteworthy findings. The mainstay of investigation revolves around critical analysis of the time-line of events leading up to anaphylaxis coupled with judicious skin testing. Skin tests have limitations with respect to unknown predictive values for most drugs/agents and therefore, knowledge of background positivity in healthy controls, test characteristics of individual drugs and the use of non-irritant concentrations is essential to avoid both false-positive and false-negative results. Specific IgE assays for individual drugs are available only for a limited number of agents and are not a substitute for skin testing. Acute serum total tryptase has a high specificity and positive predictive value in IgE-mediated POP anaphylaxis but is limited by its moderate sensitivity and negative predictive value. Planning for safe anesthesia in this group of patients is particularly challenging and consequently anesthetists need to be alert to the possibility of repeat episodes of anaphylaxis. Because of the limitations of current investigations for POP, collecting systematic data on the outcome of repeat anesthesia is valuable in validating current investigatory approaches. This paper reviews the changing epidemiology of POP with reference to the main triggers, and the investigation and outcome of subsequent anesthesia.

## Introduction

Peri-operative anaphylaxis (POP) is a serious and unpredictable iatrogenic adverse effect associated with substantial morbidity. Fatalities associated with POP are rare but occur in a significant minority of patients. In a recent audit of POP in the United Kingdom involving administration of 3 million anesthetics over a 12 months period, 10 deaths, and 40 cardiac arrests were documented in 266 patients developing anaphylaxis of grade 3–5 severity (1). While the majority of patients were successfully treated, many patients experienced anxiety associated with abandoned surgery for cancer and the physical and psychological burdens of a stay in intensive care. Of the 266 patients experiencing POP in the UK audit, 144 (54%) required critical care, with over half of this group requiring vasopressors or inotropes (1).This paper is based on a critical but narrative review, with reference to the changing epidemiology of POP and offers pragmatic, evidence-based guidance on its investigation. It should not be regarded as a systematic review.

## Epidemiology

Historically, the reported incidence of POP has varied between 1;6,000 in Norway (2) and 1:20,000 in Australia (3). However, more recent data from France (4) and the UK (5) suggests an estimated incidence of 1:10,000.These results are largely based on retrospective analysis of data on patients investigated following an episode of POP. A prospective cross-sectional study in 12 hospitals over a 2 weeks period in a single region of the UK suggests a confirmed incidence of 1:2297, with 1:353 patients fulfilling national referral criteria for anaphylaxis, suggesting significant under-ascertainment and under-reporting (6).

Alongside an increase in incidence of POP, the changing epidemiology with regard to identified underlying triggers is striking (Table 1). The traditional position of neuromuscular blockers (NMB) as the top-ranked group has now been displaced by antibiotics (5). While beta-lactam antibiotics have been a well-recognized trigger in previous series (4, 7), after NMBs, the emergence of teicoplanin in the UK as a major cause rivaling co-amoxiclav has hitherto been under-recognized and reflects its increasing use in patients with a label of penicillin allergy (5). Co-amoxiclav and teicoplanin accounted for 17.3 and 13.5% of all cases of POP, and comprised 89% of all cases of antibiotic-induced anaphylaxis. Having been absent in previous major series on POP (4, 7), the increasing importance of chlorhexidine as a major allergenic trigger is confirmed with it occupying third rank (after NMBs and antibiotics) as a cause of POP in the UK audit (5). The other new allergen described in the UK during the last decade is Patent blue dye, accounting for 5–6% of cases (5, 6). The disappearance of latex in the UK National Health Service as a cause of POP reflects the widespread adoption of latex-free measures in the operating theater and the wider hospital environment (5, 8). Despite the recognition of new triggers such as chlorhexidine and patent blue dye, successive series have identified a significant proportion of patients (27–48%) in whom no trigger is found despite comprehensive investigation (8–10). Providing advice for this group of patients is particularly challenging and highlights the limitations of current investigatory approaches.

**Table 1:** Etiology of peri-operative anaphylaxis—changing epidemiology.

	**Laxenaire et al. BJA 2001 (7)—data on 467 French patients**	**Mertes et al. JACI 2011 (4)—data on 1816 French patients**	**Harper et al. BJA 2018 (5)—data on 192 British patients**
NMBs	69.2%	58%	33.8%
Latex	12.1%	19.6%	Nil
Antibiotics-β lactams, Teicoplanin	8.0	12.8%	48.9%
Chlorhexidine	Nil	Nil	9.3%
Patent blue dye	Nil	Nil	5%
Colloids	2.7%	3.4%	1.5%
Hypnotics	3.7%	2.3%	–
Opioids	1.4%	1.6%	–

## Recognition of Anaphylaxis During Anesthesia

There are particular challenges in the recognition of anaphylaxis in an anesthetic setting because of the multiplicity of drugs administered intravenously in rapid succession and difficulty in differentiating the hemodynamic effects of anesthetic drugs from anaphylaxis. Added to this is the inherent difficulty in detecting the cutaneous features of anaphylaxis in a fully draped patient. While anaphylaxis may occur at any time during anesthesia, it is most likely to occur at induction (11). The clinical features of POP are no different to that seen when anaphylaxis occurs in a non-anesthetic setting, but severe hypotension is universally observed because of the intravenous route of administration of most drugs. Bronchospasm is less common, but is likely to be more pronounced and intractable in patients with airways disease (11).

The clinical diagnosis of anaphylaxis is aided by measurement of serial serum total tryptase in the immediate aftermath of a reaction. Acute serum total tryptase measurements correlate well with IgE-mediated reactions. In a retrospective analysis of POP, an absolute tryptase level of >15.7 mg/L or a percentage change of >141% was associated with a positive predictive value of 80% for identifying IgE-mediated anaphylaxis (8). Despite its undoubted utility it is disappointing to note that serial tryptase measurements were only undertaken in 34–67% of patients in surveys of POP in the UK (9, 12). Given its short half-life of ~2 h, tryptase levels should normalize at 24 h in virtually every patient with POP. A persistently elevated baseline tryptase (≥11.4 mcg/l) at 24 h in a patient with POP is a possible clue to an underlying clonal mast cell disorder (9, 13) or constitutionally elevated alpha-tryptase due to gene duplication associated with hyper alpha tryptasaemia syndrome (14).

## Etiologic Agents

### Neuromuscular Blockers

The major role of NMBs as a cause for POP has already been highlighted in the section on Epidemiology. Both depolarizing (suxamethonium) and non-depolarizing NMBs (benzylisoquinolines and aminosteroids) are capable of triggering POP via an IgE-mediated or non-IgE mediated pathways. Evidence for IgE-mediation is based on the demonstration of a positive skin tests and/or drug-specific IgE in serum. The ability of NMBs to trigger mast cell degranulation via the newly described MRGPRX2 receptor (15) raises the possibility that some cases of NMB anaphylaxis where evidence of IgE-mediation cannot be demonstrated are due to activation of this G-protein coupled receptor.

While the frequency of anaphylaxis with individual NMBs has not been systematically quantified, analysis of recent data from the French Pharmacovigilance Database ranked suxamethonium and rocuronium as the agents most frequently implicated in causing NMB anaphylaxis (16). Similar results were noted in the recent UK audit of POP, which identified suxamethonium, rocuronium, and atracurium (in decreasing order of frequency) as the most likely NMBs to trigger anaphylaxis (5). The lack of a history of previous exposure and the clear female preponderance in any series of NMB anaphylaxis may potentially be explained by evidence of cross-reactivity between the quarternary ammonium ions present in hair dyes, cosmetics and NMBs (17), though it would be challenging to pinpoint a precise sensitizing event. Exposure to quarternary ammonium ions via Pholcodine cough mixture coupled with laboratory evidence of potentiation of IgE antibody responses to NMB and marked epidemiological differences in the frequency of NMB anaphylaxis between Sweden (very low rate of NMB anaphylaxis) and Norway (high rate of NMB anaphylaxis), led Norway to ban pholcodine in 2007 (18). While the pholcodine hypothesis has attracted great interest, the presence of cross-reactive IgE antibodies to ammonium ions in a range of different drugs including some local anesthetics, opioids, doxycycline, and quinolones suggests multiple routes to sensitization (19, 20). A detailed analysis of the evidence around the pholcodine hypothesis was performed by the European Medicines Agency in 2011 and concluded there was insufficient evidence to recommend a Europe-wide ban (21). Since then, further data on the impact of pholcodine withdrawal on the incidence of NMB-associated anaphylaxis in Norway suggests a fall in the number of cases of anaphylaxis of grade 4–5 severity over a 6 year period (22). Interestingly, while 5 deaths due to NMB-related anaphylaxis occurred in a 2 year period immediately before and after the ban, none were recorded during the subsequent 2 years.

### Antibiotics

The place of beta-lactam antibiotics in the form of amoxicillin or co-amoxiclav as the leading antibiotic trigger for POP has recently been supplanted in the UK by teicoplanin (5). Whether this is an observation that's likely to be replicated in other countries is unclear. The choice of teicoplanin appears to be linked to patient-reported penicillin allergy, which is known to be spurious in over 90% of cases and highlights the importance of initiatives to undertake penicillin de-labeling for effective anti-microbial stewardship (23). The feasibility of undertaking pre-operative penicillin allergy de-labeling, without the need for skin testing in “low risk” patients, has recently been shown to be an effective strategy (24).

While there is increasing awareness of teicoplanin allergy amongst anesthetists, confirmation of allergy is challenging because of the limited sensitivity and lack of standardization of skin tests. In a recent series, 5 of 14 patients with either definite or probable teicoplanin-induced peri-operative anaphylaxis were negative on skin tests (25). Based on a case-series at a single institution over a 29 months period involving 18,800–19,600 patients who received teicoplanin, the rate of anaphylaxis was calculated at a frequency between 1:2088 and 1:1,655 (26).

Two other important findings have emerged from the UK national audit of POP (5). The use of a test dose in patients with a pre-operative history of penicillin allergy may itself trigger a reaction and did not reduce the severity of anaphylaxis. The use of test doses, should therefore, be discouraged. Secondly, the common practice of administering antibiotics after induction of anesthesia runs the risk of antibiotic-induced anaphylaxis being aggravated by general or neuraxial anesthesia. Changing the timing of administration of antibiotics to before induction of anesthesia would potentially be advantageous by reducing the severity of antibiotic-induced anaphylaxis, should it occur.

### Chlorhexidine

Although chlorhexidine is now well-recognized as a cause of POP occurring in 9.3 and 9.6% of cases in the UK (5) and Denmark, respectively (27), it remains a neglected allergen, frequently overlooked as a possible trigger. In the UK audit, despite it being the third most common cause of POP, chlorhexidine was only suspected as a trigger in approximately a quarter of confirmed cases. While intra-vascular (via coated central venous catheters) and or intra-mucosal exposure (via lubricating gel for urethral catheters) remains the predominant route of exposure, pure cutaneous contact may also trigger anaphylaxis (28, 29). Both skin prick testing and specific IgE measurement are reliable investigatory tools exhibiting high levels of sensitivity and specificity, exceeding 90% (27, 30). In cases where intra-dermal testing is required, it is important to use a non-irritant concentration (NICs) to avoid false-positive results (31).

Patent blue dye, used for sentinel node biopsy during cancer surgery was identified in 5% of cases in the UK survey of POP (5). A previous multi-center UK study also highlighted blue dye anaphylaxis in ~6% of cases (8). Blue dye can be potentially missed as a culprit since it is administered intradermally by surgeons and not routinely listed on drug charts by anesthetists. Mertes et al. reported a median time of 30 ± 6 min to anaphylaxis following blue dye administration and highlighted interference with pulse oximetry measurements and possible refractoriness to treatment (32). Skin tests are valuable in underpinning sensitization. Cross-reactivity with methylene blue is rare and should be tested as a potential safe alternative in those with proven patent blue allergy.

### Miscellaneous Triggers

A range of other drugs have been identified as triggers for POP including, Ondansetron, propofol, aprotinin, protamine, and ibuprofen in a small number of cases. Previous concerns about a possible increase in the risk of propofol allergy in patients with a history of egg allergy have proven to be unfounded (33). This observation coupled with the rarity of propofol allergy attests to its overall safety. Where a trigger is not identified following standard investigation, the possibility of other “hidden” allergens such as ethylene oxide should be considered (34).

## Investigation of Suspected Peri-Operative Anaphylaxis

The investigation of POP requires close attention to detail and critical analysis of the time-line of events before and after anaphylaxis. This information should be extractable from the anesthetist's letter of referral and anesthetic charts. However, in view of the illegibility of hand-written charts, completion of a pro-forma template describing the reaction is particularly important. A suitable example is the referral template recommended by the Royal College of Anesthetists in the UK (35). The timing of onset of adverse events during an episode of POP provides useful clues to the underlying trigger (Table 2).

**Table 2:** Timing of onset of anaphylaxis as a clue to underlying etiology.

**Immediately after induction**	**Intra-operative**	**At close to completion of surgery/during recovery**
Neuromuscular blockers	Antibiotics	Rectal non-steroidal anti-inflammatory agents
Antibiotics	Colloids	Opiates
Opiates	Chlorhexidine	Reversal agents
–	Dyes/contrast media	–

### Skin Testing

In view of the limited repertoire and sensitivity of currently available specific IgE assays, skin tests remain the most important investigatory tool for exploring the role of NMBs as a trigger for POP. Given the significant rate of background skin test positivity in the general population for many NMBs (36), its reliability is crucially dependent on the use of appropriate, NICs, particularly for intradermal tests. In choosing the type of skin test, comparative data on skin prick vs. intradermal tests in 212 consecutive adult patients with suspected anesthetic allergy, reveals a high degree of concordance between both routes at 93% (37). In practice, therefore, either route would be reliable for the majority of cases but reserving intradermal testing for those drugs which prove to be negative on prick testing would help reduce the risk of false-positive results with the intradermal route. The British Society for Allergy and Clinical Immunology (BSACI) recommends “neat” stock concentrations of drugs/agents alongside a 1:10 dilution (38). A positive result with the “neat” and 1:10 dilution makes it less likely to be an irritant response. More detailed, comprehensive information on optimal non-irritant concentrations for skin prick and intradermal testing is provided in a position paper by the European Network on Drug Allergy (ENDA) and the European Academy of Allergy and Clinical Immunology (EAACI) (39). Although many guidelines recommend skin testing with all NMBs (38, 40), with the aim of identifying an agent that is skin test negative, such a blanket approach increases the risk of producing false-positive results, with attendant difficulties in test interpretation, and identification of a safe alternative NMB. In addition to the index NMB, selection of a range of NMBs from both benzylisoquinoline and amino-steroid families on the basis of their least propensity to trigger histamine release is a viable alternative approach, given the high negative predictive value of NMB skin tests (9, 41). Based on the need to identify a safe NMB panel for routine and emergency anesthesia, the UK Royal College of Anesthetists has recommended the following drugs as an optimum minimum panel for testing: the suspected trigger plus suxamethonium, rocuronium, and atracurium (or cisatracurium) (42).

Despite the high negative predictive value of skin tests, anaphylaxis has been rarely documented with NMBs chosen on the basis of negative skin tests (43, 44), thus underlining the importance of utmost vigilance during repeat anesthesia.

### Drug Provocation Testing

The principles underlying the use of drug provocation tests (DPT) in the investigation of POP are no different to that in any other area of drug allergy. Whilst supervised graded DPT are regarded as a gold standard in the diagnosis of drug allergy, these are not routinely undertaken in the context of general anesthetic drugs, to NMBs in particular. They can however, be considered in selected patients following a careful risk-benefit analysis, for antibiotics, opiates/opioids, chlorhexidine, latex and local anesthetic agents to narrow down on the list of possible culprits. The use of DPT in the investigation of suspected reactions to NMBs requires even more careful consideration because of the need to use the intravenous route and the greater risk of potentially fatal anaphylaxis as opposed to an oral DPT. For the vast majority of patients experiencing NMB-associated anaphylaxis, the identification of a safe alternative following skin testing allows these patients to be safely re-anesthetized. In those rare instances where patients are skin test positive to all NMBs tested, it may be necessary to undertake an intravenous DPT in an experienced unit in an intensive care or anesthetic setting. However, it is important to emphasize the lack of consensus on undertaking DPT for NMB agents. Guidelines from ENDA/EAACI and the French Society for Intensive Care (SFAR) do not recommend performance of DPT for NMB agents (45). More recent guidelines from the Spanish Society for Allergy and Clinical Immunology echoes this stance and only recommends DPT in very exceptional circumstances (46). The current literature on DPT for NMB is confined to a single abstract from the Danish Anesthesia Allergy Center, which suggests that low dose provocation with NMB agents with negative or doubtful skin tests is safe but should still be regarded as a high risk procedure, which should be confined to highly specialized centers (47). It would be premature, therefore, to regard DPT for NMB agents as a widely accepted procedure.

### Testing for Drug-Specific IgE

Testing for specific IgE to complement but not as a substitute for skin tests is of value to a limited range of drugs. For antibiotics, drug-specific IgE assays are limited to the major and minor determinants (Penicillin V and Penicillin G) of penicillin, amoxicillin and ampicillin (48). These tests have poor predictive values and should not be requested without skin tests. The lack of a specific IgE assay to Teicoplanin poses a major problem in the light of its emergence as a major trigger for POP and the limitations of skin testing.

While specific IgE assays to various NMB (suxamethonium, rocuronium, atracurium) are available, widespread use has been hampered by the lack of systematic validation and variation in sensitivity (38.5–92%) and specificity (85.7–100%) (49). The utility of chlorhexidine-specific IgE to complement skin testing has already been discussed.

Flow cytometric assessment of basophil activation (basophil activation test; BAT) by detection of upregulated membrane markers (CD63, CD203c) in response to *ex-vivo* activation by the suspected drug is a promising technique but exhibits lower sensitivity in comparison with skin tests for NMBs (50). Whether BAT will have wider application in other areas of drug allergy remains to be determined. The logistical challenges of transporting and testing a freshly collected sample, the need for skilled personnel to run the assay coupled with restriction to a few specialist laboratories has hampered its widespread adoption. However, BAT is likely to be of value in selected patients with a compelling clinical history of anaphylaxis accompanied by an elevated acute serum total tryptase where skin tests prove to be negative (51). This view has been strengthened by a subsequent study in patients with NMB-associated anaphylaxis, where BAT exhibited a sensitivity of 77% and specificity of 76% (52).

### Timing of Investigation

**Figure 1:**
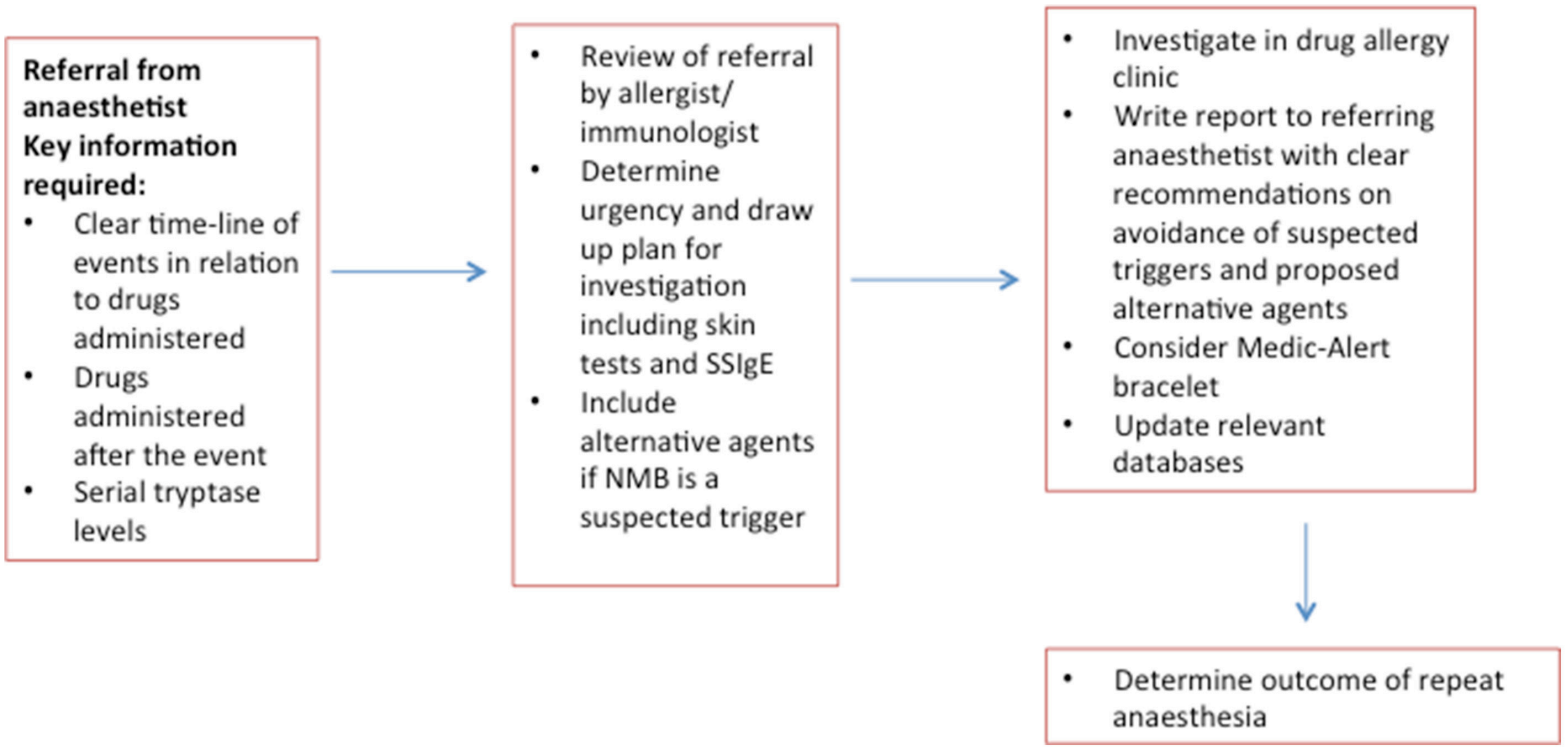
Flow chart for investigation of peri-operative anaphylaxis.

The optimal timing of investigations following an episode of POP has been the subject of debate. Based on a theoretical concern that skin testing immediately after the event may result in false-negative results due to consumption of specific IgE and mast cell refractoriness, some guidelines recommend that drug allergy investigation are performed at least 4–6 weeks after the event (53, 54). In practice, compelling clinical urgency following abandonment of cancer-related surgery does mean that some patients are skin tested within a few days of anaphylaxis. Such an approach has been shown to be valid for many patients (55, 56). However, it would be prudent to consider repeating investigations a few weeks later, if skin tests are negative and the culprit has not been identified. Figure 1 provides a flow chart of the key steps involved in the investigation of POP.

## Outcome of Repeat Anesthesia

The limitations of currently available tools for drug allergy investigation results in a failure to identify a clear trigger in a substantial proportion of patients with POP (8–10). The choice of drugs for repeat anesthesia in such patients is particularly challenging. It would be reasonable in such cases to avoid suspected triggers both in terms of its timing of administration, known cross-reactivity and propensity to trigger anaphylaxis.

Auditing the outcome of repeat anesthesia following drug allergy investigation provides quality assurance of investigatory methods and represents good clinical governance. Based on a small number of studies, the rate of repeat anaphylaxis following an index episode is estimated at 1–4% (Table 3). The emergence of undiagnosed mast cell disorders as an explanation for repeat episodes of anaphylaxis is an important observation, highlighting the need to consider bone marrow studies (61).

**Table 3:** Rate of repeat anaphylaxis.

	**Number of patients undergoing repeat anesthesia**	**Number with repeat anaphylaxis**	**%**	**Comments-identification of new triggers**
Thacker et al. (57)	57	3	5.2	NMB
Fisher et al. (58)	346	7	2.0	NMB - G,Latex- 1
Leysen et al. (41)	76	1	1.3	Inadvertent re-exposure to Chlorhexidine via coated CVC
Fisher et al. (59)	183	0	0	–
Guyer et al. (13)	47	2	4.0	Mast cell disorders
Miller et al. (9)	70	3	4.0	Mastocytosis, gelofusine, chlorhexidine
Chiriac et al. (60)	92	2	2.1	NMB

## Concluding Remarks

This review has highlighted the high stakes surrounding the investigation of peri-operative anaphylaxis and the frequent need to make recommendations based on imperfect data. Despite the limitations of drug allergy testing, it is absolutely vital that all patients with suspected POP undergo thorough investigation to avoid the morbidity and mortality associated with repeat episodes of avoidable anaphylaxis. Failure to investigate does result in repeat episodes (62) and carries significant medico-legal implications.

Investigations should be restricted to specialist allergy centers with appropriate knowledge, skill and experience in the investigation and management of drug allergy. Subject to substantial future advances in the investigatory tools available, the investigation of peri-operative anaphylaxis will remain dependent on clear documentation by anesthetists, obtaining a clear time-line of events, judicious use of skin tests and allergen-specific IgE and the exercise of astute clinical judgement.

## Author Contributions

SAM was lead author of the manuscript and was responsible for planning and writing the paper. MTK critically reviewed and co-authored the paper.

### Conflict of Interest Statement

The authors declare that the research was conducted in the absence of any commercial or financial relationships that could be construed as a potential conflict of interest.
